# Renal Tubular Dysgenesis in a Case of Fetus Acardius Amorphus

**DOI:** 10.1155/2019/5416936

**Published:** 2019-11-12

**Authors:** C. Thoeni, K. Holzer, J. Leichsenring, C. Porcel, B. K. Straub, H. P. Sinn, M. Elsaesser, A. L. Volckmar, P. Schirmacher, R. Waldherr, F. Lasitschka

**Affiliations:** ^1^Institute of Pathology, University Hospital Heidelberg, Heidelberg, Germany; ^2^Department of Laboratory Medicine and Pathobiology, University of Toronto, Toronto, Ontario, Canada; ^3^Institute of Pathology, University Hospital Greifswald, Greifswald, Germany; ^4^Institute of Pathology, University Hospital Mainz, Mainz, Germany; ^5^Department of Gynecology and Obstetrics, University Hospital Heidelberg, Heidelberg, Germany; ^6^Institute of Pathology Ludwigshafen, Ludwigshafen, Germany

## Abstract

Fetus acardius amorphus is a rare congenital malformation characterized by the lack of a functional heart, the presence of a bivascular umbilical cord, as well as a developed and organized skeletal system and partially organized inner organs. Fetus acardii mostly occur in multiple gestations. The pathogenesis of this entity is not clarified yet. It has been hypothesized that, although formation of anastomosing vessels between the co-twin and the anomalous embryo as well as reverse directed blood flow within the umbilical arteries of the weaker twin may allow sufficient blood flow to form rudimentary internal organs, it is insufficient to develop a fully functional heart. We had a case of fetus acardius amorphus, where we performed autopsy as well as routine histology assessment to identify different types of tissues. We showed that our fetus acardius amorphus demonstrated histomorphological features of renal tubular dysgenesis, confirmed by lack of proximal tubules, extramedullary hematopoiesis and increased number of smooth muscle actin positive vessels. This is a novel finding and has not been reported previously.

## 1. Introduction

Fetus acardius is a rare congenital malformation first described in the 19^th^century as acephalous acardiac monsters, presenting as an embryo lacking a heart. This is mostly observed in monozygotic twin pregnancies, but also if rarely, found in triplet gestations [1–3]. The incidence of fetus acardius has been described as 1 in 35.000 deliveries [1–12].

The main differential diagnosis is teratoma, a rare, nontrophoblastic tumor of the placenta [7, 9, 11–12]. However, there are clear distinguishing criteria between placental teratoma and fetus acardius, as reported by Fox et al. [7]. Placental teratomas are composed of unorganized parts of mature tissue, including bone and cartilage. These also lack umbilical cord tissue. Comparatively, fetus acardius consists of a usually bivascular umbilical cord, which harbors one artery and one vein attached to the placenta. There is also an organized skeletal system with the presence of a vertebral column, including ribs and pelvic bones, as well as the formation of organized internal organs with or without limb formation [7, 9, 11–12].

Fetus acardii can also be further classified into 4 subgroups. Acardius amorphus is usually detected as an ovoid mass without a head and limb formation. Acardius myelacephalus includes rudimentary limb formation. Acardius acephalus embryos do not form a head, but well developed limbs. Acardius anceps, also known as paracephalus or anceps, have a rudimentary head present. Finally, acardius acormus is the rarest variety of the acardii: this type simply has a head, but no body [8].

Here we report an interesting case of fetus acardius amorphus with typical pathology features and histomorphological features of renal tubular dysgenesis, a novel finding of this rare entity.

## 2. Case Presentation

### 2.1. Clinical History

The Fetus acardius amorphus was delivered by a 32 year-old woman, gravida 2, para 4. She presented at 29 gestational weeks for an emergency caesarian section for a twin pregnancy, because of premature rupture of membranes at the Department of Gynecology and Obstetrics, University Hospital Heidelberg, Germany. The woman gave birth to a healthy girl, as well as a boy. The boy died a few days after delivery because of a brain haemorrhage as a complication of continuous positive airway pressure (CPAP) ventilation for treatment of premature lungs. Interestingly, the mother originally presented as a triplet pregnancy early in gestation. Ultrasound examination within the first trimester of pregnancy showed the presence of three heartbeats. However, later in the pregnancy, fetal ultrasound of the second trimester only confirmed presence of two viable fetuses. At delivery, besides a placenta, an ovoid mass completely covered by skin was delivered. Both, placenta as well as the additional mass was sent for pathology evaluation to the Institute of Pathology, University Hospital Heidelberg, Germany. Informed written consent was obtained from the parents to perform autopsy and to use the fetus acardius for research purposes and publication.

### 2.2. Histology Analysis

Gross examination of the placenta demonstrated a normotrophic, monochorionic-biamnionic placenta in 22 × 10 × 3 cm of diameter and a weight of 630 g. The placenta correlated with a triplet gestation, attached with a trivascular umbilical cord. Furthermore, the placenta showed features of a physiologically circulated and appropriate-for-gestational-age developed placenta, with focal areas of dystrophic calcifications as well as focal spots of increased fibrin aggregates within the intervillous space. There was no evidence of acute or chronic inflammation or ischemia. In addition, an ovoid mass was attached to the placenta via a small pedicle, identified as Fetus acardius amorphus.

**Figure 1:**
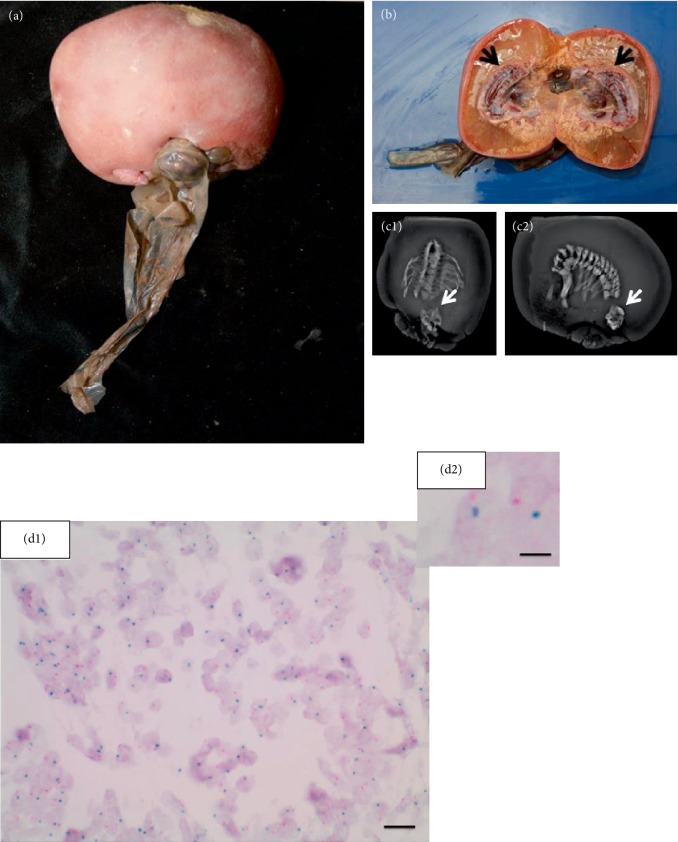
(a) Photographs of the Fetus acardius amorphus. Fetus acardius amorphus shown as a rounded ovoid mass with a small pedicle symbolizing the umbilical cord. On one surface there exists a colloidal mass, as well as a short pedicle with little hair on it. (b) The Center of Fetus acardius amorphus illustrates an organized bone structure predicted to be the vertebra column, surrounded by yellowish tissue as well as red masses. (c1 and c2) Facitron X-ray analysis of the skeletal system of the Fetus acardius amorphus. The Fetus acardius amorphus shows a complete organized vertebral column, with a rudimentary rib cage and an ovoid bone structure (white arrow) without axial organization of a head, limbs or cranial-caudal poles. (d1 and d2) CISH analysis of interphase cells within paraffin sections of male gonadal tissue using the CEN X/Y Probe. CEN X/Y Probe hybridizes on regular male interphase cells demonstrated by one red (chromosome X) and one green (chromosome Y) signal per nucleus. The Fetus acardius amorphus shows a male XY genotype. Scale bar = 20 *μ*m (D1), scale bar = 5 *μ*m (D2).

Autopsy was performed on the fetus acardius amorphus. Macroscopic examination of the fetus acardius amorphus demonstrated a mass, 10.5 × 6.5 × 7.5 cm in diameter, and covered by mature skin with sparse, superficial hair on the surface (Figure 1(a)). This mass was attached to the placenta via a 12 cm long pedicle, consisting of two major blood vessels, an artery and a vein. Furthermore a small cyst-like structure with opaque fluid inside was observed at one end of the mass, covered by intact skin and a pedicle of hair (Figure 1(a)). The center of the mass demonstrated an organized bone column with cartilage surrounded by colloidal tissue and focal red masses (Figures 1(b), 1(c1) and 1(c2)). X-ray analysis of the skeletal system with Facitron showed the presence of a complete vertebral column with the presence of a rudimentary rib cage (Figures 1(c1) and 1(c2)). At one end of the vertebral column, an ovoid bone structure was noted which could represent a rudimentary pelvis (Figures 1(c1) and 1(c2)). However, there was no axial organization of a head, limbs or cranial-caudal poles present (Figures 1(c1) and 1(c2)). After general gross examination of the anomalous embryo, representative tissue sections were fixed in 10% formalin, embedded in paraffin and stained with hematoxylin and eosin (H&E) or immunohistochemistry for histological examination. In addition, sex determination of the fetus acardius amorphus was performed on paraffin sections of gonadal tissue using the Zyto*Dot®*2C CEN X/Y Probe from ZytoVision. Sex identification analysis showed in CISH analysis a male gender with XY karyotype in interphase cells (Figures 1(d1) and 1(d2)). Detailed karyotype analysis was not performed because of insufficient tissue material.

**Figure 2:**
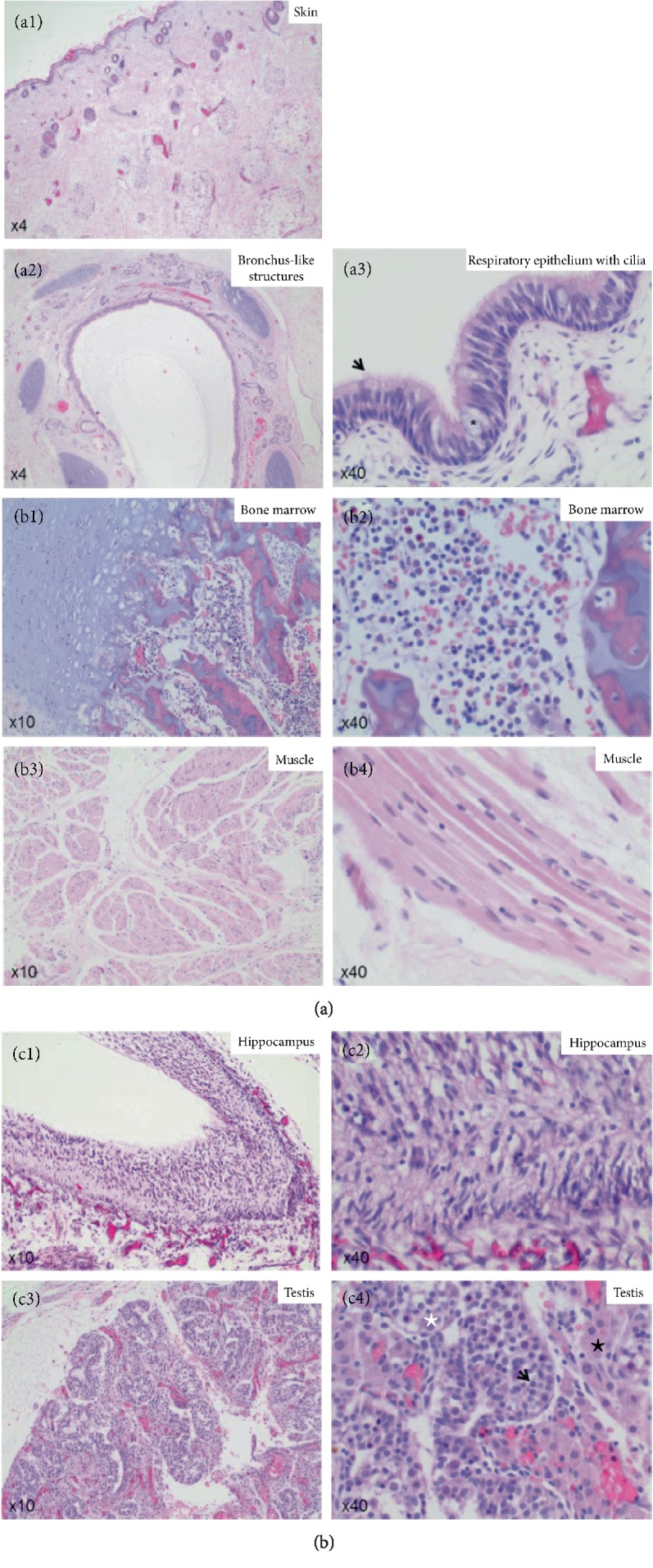
(a1): Hematoxylin and eosin (HE) staining of the skin of the Fetus acardius amorphus. Fetus acardius amorphus shows differentiated skin tissue, with a stratum corneum, an epidermis, a dermis and a subcutis. Images are shown in 4x magnification. (a2 and a3) Hematoxylin and Eosin (HE) staining of the core mass of the Fetus acardius amorphus. Fetus acardius amorphus shows organized bronchus-like structures with respiratory epithelium, goblet cells and cilia (black arrow) on the surface. Images are shown in 4x magnification (a2) and in 40x magnification (a3). (b1 and b2) Hematoxylin and Eosin (HE) staining of the core mass of the Fetus acardius amorphus. Fetus acardius amorphus shows differentiated bone and cartilage with bone marrow cells like megakaryocytes, erythrocyte precursor cells as well as myelocytes in between. Images are shown in 10x magnification (b1) and in 40x magnification (b2). (b3 and b4) Hematoxylin and Eosin (HE) staining of the core mass of the Fetus acardius amorphus. Fetus acardius amorphus shows differentiated skeletal muscle with long, shapoid myocytes with an ovoid nucleus in the center. Images are shown in 10x magnification (b3) and in 40x magnification (b4). (c1 and c2) Hematoxylin and Eosin (HE) staining of the cyst-like structure in the Fetus acardius amorphus. Fetus acardius amorphus shows differentiated neurons of the hippocampus. Images are shown in 10x magnification (c1) and in 40x magnification (c2). (c3 and c4) Hematoxylin and Eosin (HE) staining of the gonadal glands in the Fetus acardius amorphus. Fetus acardius amorphus shows gonadal glands with well-formed seminiferous tubules showing Ledyig cells between (black star), and spermatogonia (black arrow) and Sertoli cells (white star) inside. Images are shown in 10x magnification (c3) and in 40x magnification (c4).

As macroscopically organ structures could not be identified, with the exception of the vertebral column and the skin, random sections of the core of the mass were taken for detailed histology analysis. Histology analysis of the core mass showed differentiated skin tissue with all layers including epidermis, dermis and subcutis (Figure 2(a1)) as well as bronchus-like structures with respiratory epithelium with apical cilia and mucous-producing cells on top (Figures 2(a2) and 2(a3)). Furthermore, the core mass also demonstrated bone and cartilage tissue, as well as bone marrow with megakaryocytes, erythrocyte precursor cells as well as myelocytes in between (Figures 2(b1) and 2(b2)). Differentiated muscle cells with shapoid myocytes harboring an ovoid nucleus in the center were present as well (Figures 2(b3) and 2(b4)). The cyst-like structure showed brain tissue with hippocampal neurons (Figures 2(c1) and 2(c2)). Additionally parts of the urogenital tract were detected with male gonadal structures with testicles consisting of seminiferous tubules surrounded by a thin smooth muscle layer (Figures 2(c3) and 2(c4)). Inside the seminiferous tubules, spermatogonia as well as Sertoli cells were present, Leydig cells were embedded between the semiferous tubules, respectively (Figures 2(c3) and 2(c4)).

**Figure 3:**
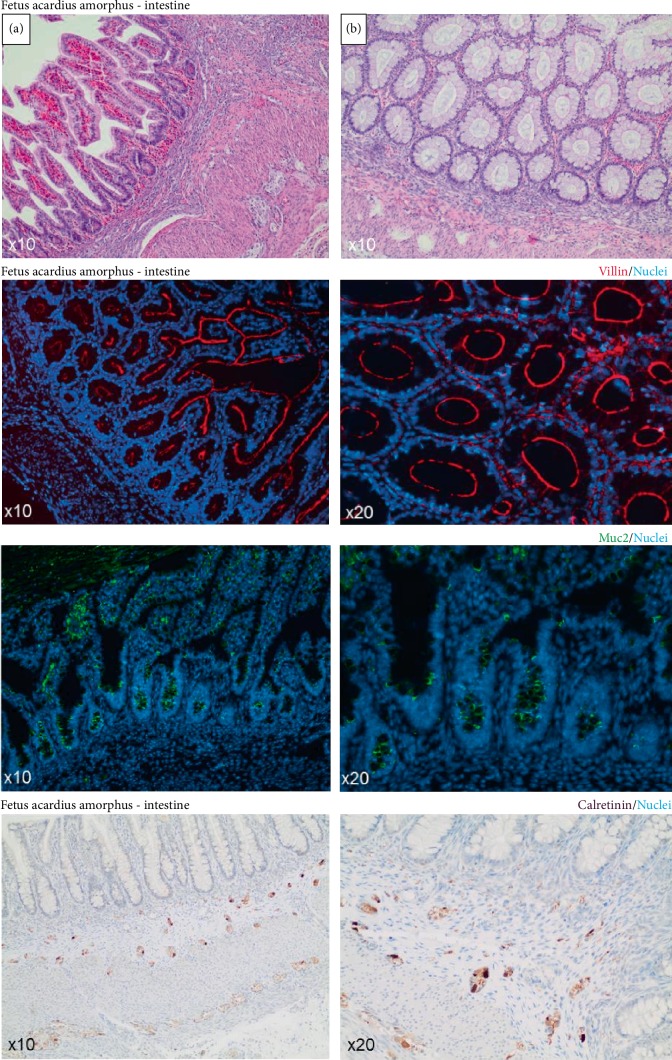
Hematoxylin and Eosin (HE) staining and immunohistochemistry staining for villin, mucin 2 and calretinin in the intestine of the Fetus acardius amorphus. Villin is stained in red (ALEXA 568), mucin 2 in green (ALEXA 488) and nuclei in blue (Hoechst). Fetus acardius shows differentiated small (a) and large (b) intestine with positive apical villin staining (red) marking the intestinal brush border in cells of the crypt and villi and mucin 2 (green) positive Goblet cells in crypts and villi. Ganglion cells of the Auerbach and Meissner Plexus were also present shown by positive Calretinin staining. Images are shown in 10x and in 20x magnifications.

Moreover, within the focal red masses parts of the gastrointestinal tract with small as well as large intestine were identified (Figures 3(a) and 3(b)). The intestine was organized in a crypt villous axis, and enterocytes showed an apical brush border strongly positive for the brush border protein villin in both, crypts and villi (Figure 3). In addition, Goblet cells were identified with positive mucin 2 stains (Figure 3). Ganglion cells positive for Calretinin were detected within the Meissner and Auerbach Plexus (Figure 3).

**Figure 4:**
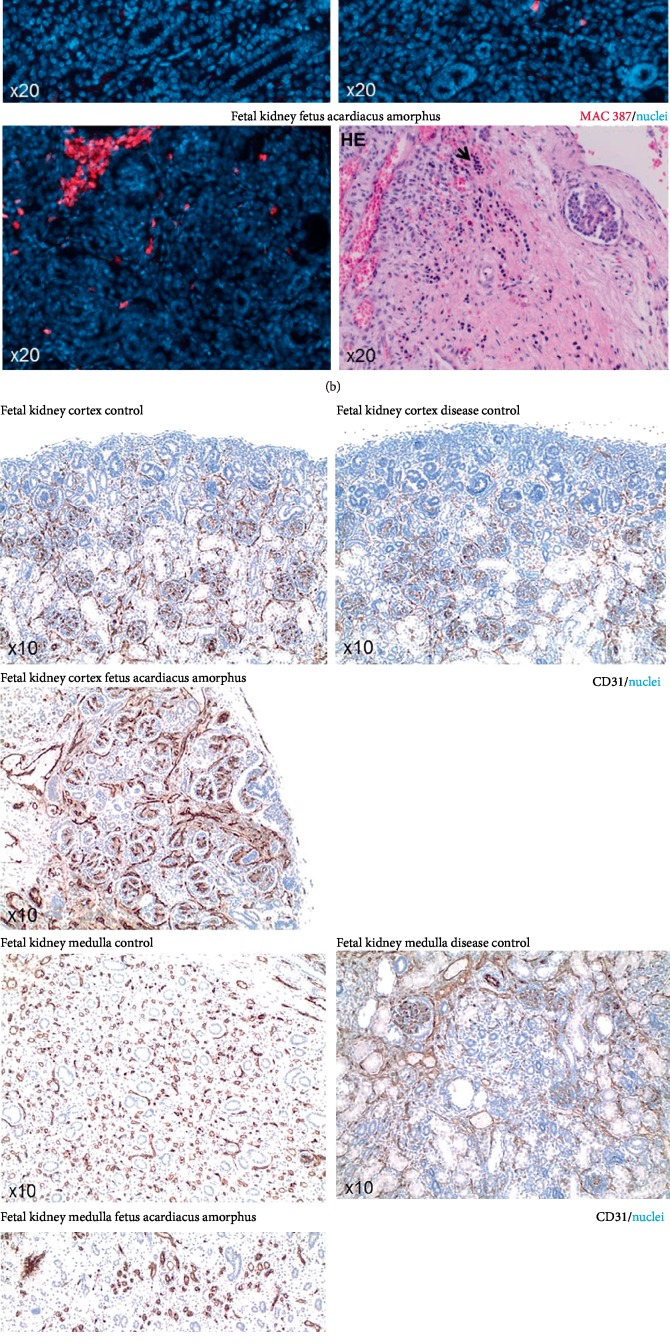
(a) PAS staining and immunohistochemistry staining for villin of fetal renal tissue. In immunohistochemistry, villin is stained in red (ALEXA 568) and nuclei in blue (Hoechst). Fetus acardius shows differentiated kidney with cortex and medulla, but lack of proximal tubules as shown by negative villin staining. Additionally, distal tubules of the fetus acardius amorphus show mild nephrocalcinosis (white star). PT (proximal tubule), G (glomerulus). Images are shown in 10x, 20x and 40x magnifications. (b) Hematoxylin and Eosin (HE) staining and immunohistochemistry staining for MAC 387 staining in fetal renal tissue. MAC 387 positive cells were detected in the interstitial tissue of the kidney in the Fetus acardius amorphus, those cells are marked with a black arrow in the Hematoxylin and Eosin (HE) staining labeling erythrocyte precursor cells. MAC 387 is stained in red (ALEXA 568), nuclei stained with in blue (Hoechst). Images are shown in 20x magnification. (c) CD31 immunohistochemistry staining in fetal renal tissue. CD31 (brown) is staining vessels in the renal cortex and medulla, nuclei are stained with hematoxylin in blue. Increased number of CD31 positive vessels is detected, in particular in the renal cortex of the fetus acardius amorphus.Images are shown in 10x magnification. (d) Smooth muscle actin (SMA) staining in fetal renal tissue. Increased number of SMA positive vessels in particular the cortex of the fetus acardius amorphus. SMA is stained in green, nuclei stained with Hoechst in blue. Glomeruli are marked with a white star, tubules with white arrows. Images are shown in 10x magnification.

Intriguingly, the most curious finding of this case of Fetus acardius amorphus was the abnormalities within the renal tissue (Figure 4). Indeed, the kidney of the Fetus acardius amorphus demonstrated differentiated glomeruli, but a complete lack of proximal tubules, confirmed by PAS staining as well as villin immunohistochemistry (Figure 4(a)). Findings of the fetus acardius amorphus were compared to a fetus without any evidence of abnormalities (control) and a fetus with bilateral renal hypoplasia (disease control). Both control cases were aborts from women between 20 and 25 weeks of gestational age. The control case was an intrauterine fetal death without finding an underlying maternal or fetal cause of death; the disease control was a fetus with intrauterine death because of bilateral renal hypoplasia. In both control cases, proximal tubules were present (Figure 4(a)).

In addition, within the medulla, clusters of erythrocyte precursor cells were detected, usually just found in the spleen and liver as a sign for extramedullary hematopoiesis (Figure 4(b)). Immunofluorescence of those clusters of cells showed positivity for the myeloid marker MAC 387, identifying these cells as erythrocyte precursor cells (Figure 4(b)). This was interesting because, in general, extramedullary hematopoiesis in the kidney occurs if oxygenation is impaired. Therefore, immunohistochemistry for investigating angiogenesis within the kidney was performed. Stainings of vessels with CD31 showed particularly in the renal cortex of the Fetus acardius amorphus increased presence of CD31 positive vessels, mainly within the interlobular area (Figure4(c)). Interestingly, those vessels were strongly positive in immunofluorescence stains for smooth muscle actin (Figure 4(d)). Those SMA positive vessels also seemed to have a higher degree of dilation, when compared to both controls (Figure 4(d)). However, the vessel wall did not seem to appear thicker in the case of Fetus acardius amorphus when compared to both controls. Those findings, the lack of proximal tubules, extramedullary hematopoiesis, and increased number of smooth muscle actin positive vessels are also commonly found in cases of tubular dysgenesis, although in tubular dysgenesis, arterial wall thickening and disorganization of interlobular and afferent arteries has been additionally described, which was not identified in our case of Fetus acardius amorphus. Taken together, our case of fetus acardius amorphus showed histomorphological features of tubular dysgenesis, which could occur secondary to major cardiac malformations, or the lack of a functional heart, as is the case in this presentation of Fetus acardius amorphus.

## 3. Discussion

Fetus acardius belongs to the class of rare congenital malformations, occurring in one of 35.000 deliveries [1–12]. The main characteristics of a fetus acardius are the absence of a functional heart and the existence of an umbilical cord, mainly bivascular, as well as organized bone and cartilage and rudimentary internal organ formation [7]. Fetus acardii are classified into 4 subgroups defined upon the presence and/or lacking of a head, limbs and a body [8]. As all variants of Fetus acardii, including our case, which lacks a functional heart, the presence of a co-twin is always required for providing sufficient blood flow for both embryos [9–12]. Therefore this condition is mainly found in monozygotic, monochorionic twins and rarely in triplet gestations. The aetiology and pathogenesis of Fetus acardius are still not clearly defined. As a main theory, it has been hypothesized that via formation of anastomosing vessels between the co-twin and the anomalous embryo as well as reverse directed blood flow within the umbilical arteries of the weaker twin, sufficient blood flow might be existing to form rudimentary internal organs, but insufficient to develop a fully functional heart [1–12]. Furthermore, the deficiency of oxygen and nutrients in the blood of the anomalous embryo causes a malnutrient and anoxic environment that might result in impaired development [1–12]. Another theory might be that compression of the embryo at the embryonic disc stage could cause failure in embryonic development [1–12]. Intriguingly, so far no specific genetic variants or chromosomal abnormalities have been described in the development of fetus acardii [12].

Furthermore, it is of importance to exclude the main differential diagnosis of placental teratoma, a very rare tumor of the placenta [7, 9, 11, 12]. The main distinction criteria of placental teratomas from fetus acardius are that fetus acardius demonstrates an umbilical cord as well as an organized skeletal development including a vertebral column and rudimentary formed internal organs, while in teratomas of the placenta just remnants of disorganized mature tissue, mainly bone and cartilage, are present [7]. It has been reported that, in certain cases, it has been difficult to distinguish between placental teratomas and fetus acardius according to those criteria. Helpful facts may be to consider clinical information about pregnancy course and history as well as early evidence of multiple gestations besides the pathology features [12]. Moreover, incorporating functional genetic studies of placental teratomas as well as fetus acardii would be of interest to shed more light on the pathogenesis and development of both, placental teratomas as well as fetus acardii. This may also identify genetic hallmarks, which are specific just for one of both entities, resulting in a clear definition of either placental teratoma or fetus acardii to finally confirm the diagnosis.

Our case of Fetus acardius amorphus includes the typical hallmarks of an organized skeletal system and rudimentary formed organs. Among those, the most differentiated tissues were skin, intestine and rudimentary kidney. Surprisingly, a novel finding in our case of Fetus acardius amorphus was the occurrence of histomorphological features of renal tubular dysgenesis characterized by lack of proximal tubules, extramedullary hematopoiesis and increased angiogenesis, described as a consequence of major cardiac malformations and decreased perfusion of kidneys *in utero*. In addition, a common finding in tubular dysgenesis is characteristic arterial wall thickening and disorganized interlobular and afferent arteries, a finding, which was not clearly identified in our case of Fetus acardius amorphus [13]. We just noted an increased number of dilated, smooth muscle actin positive vessels, particularly in the renal cortex, implicating increased angiogenesis and hyperperfusion in the kidney of the fetus acardius amorphus. In conclusion, we report features of renal tubular dysgenesis as a novel finding in a case of Fetus acardius amorphous.

## Acklowledgments

The authors thank Karoline Fiedler for proofreading, Jutta Scheuerer, Jessica Trost and Sarah Meßnard for technical assistance, Prof. Dr. Herpel and her team of the NCT Tissue Biobank for providing tissue, John Moyers for taking photographs and the parents of the fetus acardius case as well as both control cases who give consent for the study as regulated by the ethical guidelines of the NCT Tissue Biobank and defined by the local ethics committee (ethical vote 206/05).
